# Biased Opioid Ligands

**DOI:** 10.3390/molecules25184257

**Published:** 2020-09-16

**Authors:** Abdelfattah Faouzi, Balazs R. Varga, Susruta Majumdar

**Affiliations:** Center for Clinical Pharmacology, St. Louis College of Pharmacy and Washington University School of Medicine, St. Louis, MO 63131, USA; abdelfattah.faouzi@stlcop.edu (A.F.); balazs.varga@stlcop.edu (B.R.V.)

**Keywords:** G-protein bias, arrestin recruitment, opioid receptors, respiration, mitragynine, analgesia

## Abstract

Achieving effective pain management is one of the major challenges associated with modern day medicine. Opioids, such as morphine, have been the reference treatment for moderate to severe acute pain not excluding chronic pain modalities. Opioids act through the opioid receptors, the family of G-protein coupled receptors (GPCRs) that mediate pain relief through both the central and peripheral nervous systems. Four types of opioid receptors have been described, including the μ-opioid receptor (MOR), κ-opioid receptor (KOR), δ-opioid receptor (DOR), and the nociceptin opioid peptide receptor (NOP receptor). Despite the proven success of opioids in treating pain, there are still some inherent limitations. All clinically approved MOR analgesics are associated with adverse effects, which include tolerance, dependence, addiction, constipation, and respiratory depression. On the other hand, KOR selective analgesics have found limited clinical utility because they cause sedation, anxiety, dysphoria, and hallucinations. DOR agonists have also been investigated but they have a tendency to cause convulsions. Ligands targeting NOP receptor have been reported in the preclinical literature to be useful as spinal analgesics and as entities against substance abuse disorders while mixed MOR/NOP receptor agonists are useful as analgesics. Ultimately, the goal of opioid-related drug development has always been to design and synthesize derivatives that are equally or more potent than morphine but most importantly are devoid of the dangerous residual side effects and abuse potential. One proposed strategy is to take advantage of biased agonism, in which distinct downstream pathways can be activated by different molecules working through the exact same receptor. It has been proposed that ligands not recruiting β-arrestin 2 or showing a preference for activating a specific G-protein mediated signal transduction pathway will function as safer analgesic across all opioid subtypes. This review will focus on the design and the pharmacological outcomes of biased ligands at the opioid receptors, aiming at achieving functional selectivity.

## 1. Introduction

G-protein coupled receptors (GPCRs) are a 7 transmembrane-spanning evolutionary conserved superfamily that has been well described in the literature and the subject of extensive studies for the last couple of decades [1,2]. They can bind to a very large variety of signaling molecules and consequently play an incredible array of function in the human body [2]. It has been estimated that more than one-third of the drugs currently marketed interact with GPCRs [3].

Among this class of molecules, the opioid receptors represent one of the biggest targets in modern medicine [4]. Indeed, the current “opioid crisis” is one the biggest challenges in therapeutics, especially in the United States [5]. To this day, several questions about these receptors remain unanswered, which is the main reason behind the inability to treat addiction efficiently and/or synthesize pain-relieving agents devoid of side effects. This prompts further research to specifically understand the entire physiology and mechanisms of action linked to the opioid receptors and GPCRs in general. This distinct class of molecules encompasses the MOR (µ-opioid receptor), KOR (κ-opioid receptor), DOR (δ-opioid receptor), the NOP (nociceptin opioid receptor) and can be activated by both endogenous or exogenous opioids ligands, with morphine being the prototypic agent [2,6]. Upon activation, GPCRs are known to experience conformational changes, subsequently leading to different corresponding signaling pathways [2,6]. GPCRs transduction signaling is dependent on the receptor-mediated activation of heterotrimeric G proteins, which are composed of three subunits, Gα, Gβ, and Gγ respectively [1]. When bound to GDP, Gα associates with the Gβγ dimer in order to form the inactive heterotrimer. Receptor activation promotes the engagement of the GDP-bound heterotrimer that accelerates GDP dissociation from Gα. Subsequently, the Gα subunit undergoes conformational changes resulting in the dissociation of the Gα and Gβγ subunits. Both subunits have been shown to modulate the activity of different downstream effector proteins with Gα targeting effectors including adenylyl cyclases or cGMP phosphodiesterase while Gβγ recruits GRKs to the membrane and regulates G-protein-coupled phosphoinosite 3 kinase (PI3K) or mitogen-activated protein kinases (MAPK). The final step of this cycle consists of a GTP to GDP hydrolyzation process promoted by the Gα subunit GTPase intrinsic activity, which then re-associates with Gβγ to complete the G-protein activation circle [1].

The ability to elicit a preferential signaling pathway depending on the spatial molecular rearrangement towards an orthosteric ligand is called “biased agonism” or “functional selectivity” [7,8,9,10]. This concept remains unclear and is very disputed within the scientific community in terms of reaching general consensus about its exact definition. Nonetheless, the discovery of such phenomenon has been of tremendous interest in the fields of drug discovery, academia, and the drug industry [11]. Most importantly, it offers a therapeutic alternative to conventional opioid analgesics (in particular to the ones targeting the MOR such as morphine) which are well known for inducing several adverse reactions such as tolerance, dependence, constipation in addition to respiratory depression and addiction.

**Figure 1:**
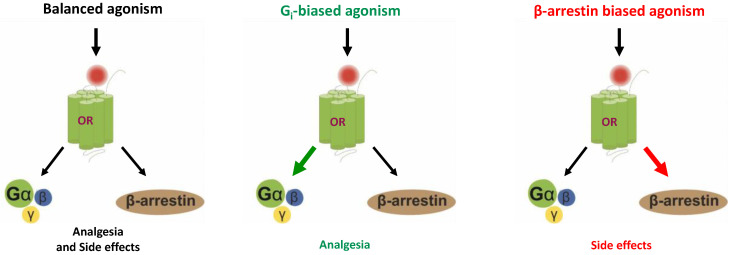
Functional selectivity correlation of opioid agonists. Ligands not recruiting β-arrestin 2 at all opioid subtypes are proposed to dissociate subtype selective adverse effects from its pain-relieving properties. In the case of the μ-opioid receptor (MOR), biased ligands will have less tolerance. For KOR, ligands should have less sedation and anhedonia. Biased DOR agonists should separate convulsions from analgesia while role of biased NOP receptor ligands is less well characterized, although it is possible that memory impairment, sedation, and hypothermia may be dissociated.

It is now well established that the activation of opioid receptors triggers two main transducing pathways with more or less preference: the β-arrestin 2 or/and the G-protein pathway [12,13]. The β-arrestin 2 (ubiquitously expressed) regulates opioid receptors signaling through desensitization and internalization, while the G-protein pathway is the “classical” signaling route and will promote different effects depending on the opioid receptor subtype, including analgesia [7,14]. Additionally, it has now been reported that biased opioid receptors ligands induce conformational changes of the receptor, activating a specific signaling pathway. In fact, several structural studies showed that G protein-biased ligands stabilize a certain opioid receptor conformation distinct from the conformation stabilized by β-arrestin biased ligands, which correlates with an equilibrium between the active and inactive states of the receptor and dictates the selective engagement between G-protein and β-arrestin [1,15,16]. These studies provide invaluable insights into the binding mode of these proteins and shed light on which specific residues are involved in these processes. However, further investigations are required in order to truly comprehend this phenomenon which is drawing even more growing interest to this field [17]. The concept of functional selectivity in its simplest form is represented in Figure 1.

The primary aim of this article will be to provide a non-exhaustive list of biased agonists of all the opioid receptors with an understanding of the limitations and advantages both in vitro and in vivo that they can provide. Also, we will focus on the significance and potential future avenues for the development of biased ligands and their analogs targeting the opioid receptors.

## 2. MOR Biased Agonism

For more than 20 years now, the synthesis of biased agonists targeting the MOR has been considered as a credible strategy in order to mitigate analgesia from the classical opioids side effects. It started in 1999, when Bohn et al. demonstrated an improved antinociceptive effect of morphine in β-arrestin 2 (or arrestin-3) KO mice [18]. Determining the extent of β-arrestin 2 involvement in the development of side effects was the focus of follow-up studies, which demonstrated reduced gastrointestinal, respiratory depressant effects, and tolerance of morphine in β-arrestin 2 KO mice [19,20]. These results were recapitulated in mice by knocking down β-arrestin 2 with antigene RNA and siRNA. While antinociception was found to be prolonged, tolerance was diminished [21,22].

**Figure 2:**
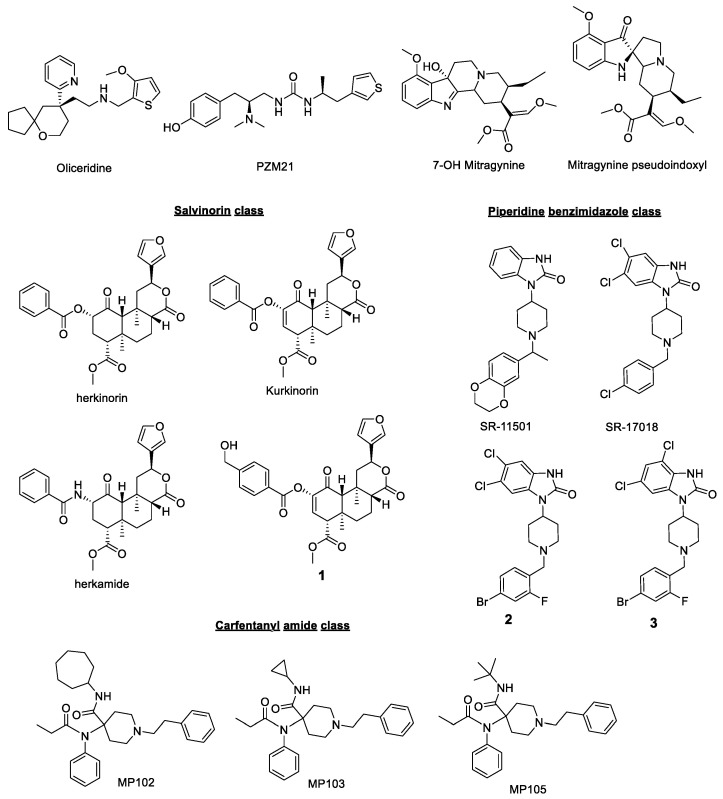
Structures of MOR biased ligands reported having different levels of β-arrestin 2 recruitment.

These results were significant in the opioid field and created a whole new approach in the design of G protein-biased agonists, thought to be “better opioids” with an improved safety profile targeting the MOR (Figure 2 shows a list of these ligands targeting MOR). In all cases, DAMGO ((D-Ala(2)-mephe(4)-gly-ol(5))enkephalin) is considered as the standard balanced agonist as the comparator, unless otherwise stated. A non-exhaustive list of MOR biased ligands with potency and efficacy at the G-protein and the β-arrestin2 pathways is shown in Table 1.

**Oliceridine/TRV130**

[(3-methoxythiophen-2-yl)methyl]({2-[(9R)-9-(pyridin-2-yl)-6-oxaspiro[4.5]decan-9-yl]ethyl)amine), also known as TRV130 or Oliceridine was the first G-protein biased ligand developed by the pharmaceutical company Trevena in 2013 [23]. The discovery of this agent was the result of a high-throughput screening (HTS) of their chemical library followed by further optimization for potency, ligand bias, and selectivity toward the MOR. In their initial study, the authors found that TRV130 was a G-protein partial agonist of the MOR which exhibited a 3-fold preference for the G-pathway over the β-arrestin 2, relative to morphine and fentanyl [23]. In HEK cells, Oliceridine was uncovered to be more potent for G-protein stimulation (EC_50_ = 8 nM vs. 50 nM for morphine) but less active on β-arrestin 2 recruitment compared to morphine (14% of morphine efficacy). TRV130 signaling has been studied by other groups too (See Table 1 for details). Perhaps due to its ability to act as a G-protein weak partial agonist, Oliceridine also displayed a very safe side effects profile in vivo with reduced constipation and respiratory depression in these initial findings. Additionally, the measurements of antinociception proved that this agent was a powerful analgesic in rodents in the hot plate assay for mice (ED_50_ = 0.9 mg/kg vs. ED_50_ = 4.9 mg/kg for morphine) and in several assays for rats such as the tail-flick or the rat hindpaw incisional pain model.

Consequently, Oliceridine advanced through several phases of clinical trials, was outvoted in 2018 initially, and was finally approved by the U.S. Food and Drug Administration (FDA) in 2020 [35].

In earlier clinical trials, it produced opioid-like subjective effects in humans, suggesting abuse liability [36] consistent with recent reports in the literature on TRV130 in rodents shows mixed results with the drug showing constipation, addictive properties, and tolerance similar to classical MOR agonists in rodents while also showing less signs of somatic withdrawal, raising doubts about the drug’s safety profile [37,38]. However, with the recent FDA approval, the potential of G-biased agonism can now finally be tested in humans and findings may help the field to either call it a day on MOR biased agonsim or push for the development of additional biased agonists.

**PZM21**

1-[(2S)-2-(dimethylamino)-3-(4-hydroxyphenyl)propyl]-3-[(2S)-1-(thiophen-3-yl)propan-2-yl]urea, also known as PZM21 was the first in class structure-based discovered G-protein biased agonist of the MOR by a group of scientists from the University of Stanford, UCSF, UNC-Chapel Hill and, Friedrich-Alexander-Universität Erlangen-Nürnberg in 2016 [24]. Over 3 million molecules were computationally docked against the inactive structure of MOR which led to the identification of PZM21 as a non-prototypical potent G_i_ activator at MOR with minimal β-arrestin 2 recruitment (see Table 1 for PZM21 signaling studies by other groups). PZM21 also showed great selectivity for MOR over other 330 other off-targets [24]. The investigations on PZM21 as a potential analgesic also showed long-lasting potent antinociception in the hot plate and formalin injection assays, but, curiously, no effect was detected in the tail-flick assay. It is well-described in the literature that the (mice or rat) tail-flick test is a measure of spinal reflexes response which would mean that PZM21 only triggers a supraspinal effect in vivo [39]. Moreover, PZM21 analgesia was completely abolished in MOR KO mice which proved that the effect was MOR-mediated. Finally, the authors showed that PZM21 did induce constipation but to a lower extent compared to morphine and, impressively, did not cause respiratory depression, conditioned place preference (CPP), nor significantly increased locomotion.

In contrast, a follow-up study from another group in 2018 proved that although PZM21 was indeed a potent analgesic, it caused significant, rapid, and persistent respiratory depression in C57/BL and CD-1 mice when injected intraperitoneally or subcutaneously [27]. This effect was comparable to that of a 10 mg/kg equianalgesic dose of morphine.

Another recent study confirmed that PZM21 caused long-lasting dose-dependent antinociception and did not induce reward- and reinforcement-related behavior [40]. However, the authors showed that PZM21 led to the development of antinociceptive tolerance and naloxone-precipitated withdrawal symptoms after multiple administrations. They also showed that pretreatment with PZM21 could increase morphine-induced antinociception and attenuate the expression of morphine reward. In contrast to the initial investigations of this agent, they expressed concerns about PZM21 clinical applications.

Intrathecal opiate use with drugs like morphine is usually impacted by the risk of producing space-occupying intrathecal masses through Mas-related G-protein coupled receptor (MRGPR) signaling. A recent study by the Yaksh group shows that PZM21 does not produce mast cell degranulation or activation of fibroblasts because it doesn’t activate MRGs, unlike morphine, suggesting that MOR biased ligands could be useful for intrathecal therapies [41].

**7-OH mitragynine/Mitragynine pseudoindoxyl**

The psychoactive plant *Mitragyna speciosa* (also known as “kratom”) has been used in Southeast Asian traditional medicine as a tea or directly chewed for centuries in order to treat a large range of pathologies due to its opioid properties and stimulant-like effects [42]. Numerous alkaloids have been isolated from this plant, including the primary constituent indole-scaffold based mitragynine and the oxidized derivative 7-hydroxymitragynine (7-OH). This latter compound has been well documented in the literature and a series of papers from a group of Chiba University researchers reported its activity as a MOR selective agonist which produced a full antinociceptive effect in the mouse-tail flick at a 5 mg/kg or 10 mg/kg concentration when administered subcutaneously or orally, respectively [43]; an effect antagonized by naloxone injection. Classical opioid-like side effects were reported in the same study, such as withdrawal, constipation (albeit less than morphine), and tolerance [44,45]. Another study from 2016 looked more closely at the pharmacology of mitragynine and 7-OH [28]. The authors proved that both agents were G_i_-biased partial agonist of the MOR which did not recruit β-arrestin 2. Moreover, the authors claimed that the very weak signal obtained in β-arrestin 2 recruitment assays did not allow for bias quantification. The G-protein bias of 7-OH has been replicated independently by two groups (See Table 1) [25,29]. Another collaborative effort between the Memorial Sloan Kettering Cancer Center and the University of Florida in the same year also proved that 7-OH was 5 times more potent than morphine when administered subcutaneously [29]. Furthermore, this study drew attention to another oxidized derivative from mitragynine, namely mitragynine pseudoindoxyl (MP) [29]. The authors showed that MP is a high affinity agonist at MOR in [^35^S]GTPγS assays while it shows no β-arrestin 2 recuitment. In vivo in mice, MP was at least 1.5 and 3-5 times more potent than morphine after intracerebroventricular and subcutaneous administration, respectively, in different strains of mice (CD1, C57BL/6, and 129Sv6). Side effect analyses additionally emphasized the development of analgesic tolerance but showed that it was far slower compared to morphine (29 days vs. 5) and the same observation could be made with constipation and dependence. Finally, this agent failed to show either aversive or rewarding effect when tested in the conditioned place preference paradigm. Finally, a follow-up study from 2019 proved that mitragynine is converted to 7-OH by cytochrome P450 through an hepatic metabolism-dependent mechanism and that high concentration of this agent could be retrieved in the plasma/brains of mice, which explains its analgesic properties [46]. The study also conclusively showed 7-OH analgesic actions being MOR-dependent and KOR- and DOR-independent using opioid subtype KO mice. Recent studies showed that 7-OH is self-administered in rats like other MOR modulators suggestive that bias at MOR may not dissociate addiction from analgesia [47].

**Herkinorin/Kurkinorin**

Methyl(2*S*,4a*R*,6a*R*,7*R*,9*S*,10a*S*,10b*R*)-9-(benzoyloxy)-2-(furan-3-yl)-6a,10b-dimethyl-4,10-dioxodode-cahydro-2*H*-benzo[*f*]isochromene-7-carboxylate and methyl (2*S*,4a*R*,6a*R*,9*S*,10a*R*,10b*R*)-9-(benzoyloxy)-2-(furan-3-yl)-6a,10b-dimethyl-4,10-dioxo 1,4,4a,5,6,6a,9,10,10a,10b-decahydro-2*H*-benzo[*f*]isochromene-7-carboxylate, more commonly known as Herkinorin and Kurkinorin, respectively, are analogs of the natural product Salvinorin A described as an agonist of the KOR [48]. The SAR of Sal A was vastly studied and subsequent chemical modification allowed for the synthesis of other opioid receptors ligands, including herkinorin and kurkinorin [49]. Interestingly, these agents were described as the first non-nitrogenous MOR agonists, and displayed strong affinity toward the MOR (K_i_ = 1.2 nM and 40 nM for Kurkinorin and Herkinorin, respectively) [50]; both compounds did not recruit β-arrestin 2 while Herkenorin in addition did not promote receptor internalization [30,48]. The arrestin recruitment of Herkenorin at MOR has been recently been challenged (Table 1) [24]. Follow up studies from the Prisinzano group yielded several analogs on the Sal A template which were then biologically tested in vitro and in vivo [32]. One particular analog of interest was herkamide, where the phenyl ester was replaced by a phenyl amide substituent. Herkamide in contrast to herkenorin robustly recruited β-arrestin 2 and internalized MOR. Similar studies on Kurkinorin proved that it was a G_i_-protein biased agonist of the MOR, which demonstrated potent centrally-mediated antinociception in the hot water tail-flick test in male B6-SJL mice equal to morphine while Herkinorin acted as a peripherally restricted analgesic (active in the rodent formalin test but no activity detected in the tail flick-assays) [31]. Pre-treatment with naloxone blocked the antinociceptive effect of Kurkinorin, proving that the effect was indeed MOR specific. Of note, the authors raised questions about the CNS penetrating potential of Kurkinorin compared to Herkinorin given the strong similarities in the chemical scaffolds, cLogP, and PSA of both entities. Side effects profiling showed that Kurkinorin induced tolerance but significantly less compared to morphine. Similarly, using the rotarod behavioral assay, it was shown that Kurkinorin impaired motor coordination but considerably less compared to morphine, while Herkinorin had no effect, which was in accordance with the non-CNS penetrating ability of this agent. Finally, the addition of a lesser rewarding effect of Kurkinorin compared to morphine (assessed through condition place preference paradigm) highlights these agents as very promising alternatives to the classical opioid therapies, but further studies need to be undertaken in order to shed light on the exact mechanism of action and mitigate centrally-induced side effects. A recent report from the same group showed an analog of Kurkinorin with a p-CH_2_OH substitutent (Methyl (2S,4aR,6aR,7R,10aR,10bR)-2-(furan-3-yl)-9-((4- (hydroxymethyl)benzoyl)oxy)-6a,10b-dimethyl-4,10-dioxo-1,4,4a,5,6,6a,7,10,10a,10bdecahydro-2H-benzo[f]isochromene-7-carboxylate, **1**, Figure 2 and Table 1) retaining the G-protein bias at MOR and low analgesic tolerance potential of the parent template. This compound was 100-fold more potent over morphine both in vitro and in vivo [32].

**Piperidine benzimidazoles**

In 2017, a collaboration from two groups of the Scripps Research Institute led to the development of a series of substituted piperidine benzimidazole with high agonistic affinity for the MOR [33]. This very thorough study explored the SARs of these agents which resulted in the identification of several analogs with high G-protein bias and showed that halogen substituents (grafted on different parts of the scaffold) favored MOR conformations that promote robust [^35^S]GTPγS binding while disfavoring β-arrestin 2 signaling. Most notably, SR-17018, SR-15098, and SR-15099 were completely inactive in the β-arrestin 2 recruitment assay, which was an issue in order to quantify bias factors as fairly stated by the authors. As such, and in order to make sure none of the synthesized analogs were merely potent partial agonist, the quantification model was modified accordingly and the authors looked at the ability of these agents to block a stimulatory 10 μM dose of DAMGO. In addition, G-protein activation was measured through two different assays: forskolin-stimulated cAMP accumulation in CHO-hMOR cells and stimulation of [^35^S]GTPγS binding in membranes. As a result, G-protein bias was preserved for all these derivatives with the exception of SR-11501 which appeared to be a balanced agonist of the MOR. In vivo studies in mouse brainstem confirmed the activity of the piperidine benzimidazoles as G-protein agonists of the MOR with SR-11501 being the least potent (which was in agreement with previous in vitro assays) while no activity was detected in MOR KO mice, proving the high selectivity of the whole series. Noteworthy, the authors emphasized that bias quantification can differ significantly when the inhibition of cAMP stimulation is used as a measure of G-protein signaling. The SR MOR agonists were also uncovered to be long-lasting, brain penetrant (after intraperitoneal administration), and to promote potent antinociception in both the hot plate and warm water tail withdrawal assays equal to morphine and fentanyl. Interestingly, when these agents were tested for respiratory depression, the derivatives with the highest G-protein bias displayed the lowest respiratory depression, below to that of morphine at a same equi-antinociceptive dose. Finally, this work showed that bias factors can correlate with therapeutic window, and a strong linear correlation was established between these two features. A recent report, however, challenges the bias hypothesis as a mechanism for the lower respiratory depression potential of SR-17018 compared to morphine (also see Table 1). In 2018, a follow-up study from the same groups looked at iteratively optimizing and expanding the SARs of this series (with modification of the substituents and the central ring size) while analyzing bias factors, which the authors referred to as “bias-focused SAR study” [34]. This work also shed light on other DMPK parameters such as cytochrome P450 inhibition, suitable half-life, and even microsomal stability. As a result, they managed to identify structural features such as the presence of halogens and a central piperidine which positively impacts bias and safety profiles. As in the initial Cell paper study, a pair of interesting compounds were identified. Compound **2** with di-Cl groups ortho to each other (5,6-Dichloro-1-(1-(4-bromo-2-fluorobenzyl)piperidin-4-yl)-1*H*-benzo[*d*]imidazol-2(3*H*)-one) showed high G-protein bias (β-arrestin 2 E_max_ = 12%) compared to compound **3** where di-Cl groups (4,6-Dichloro-1-(1-(4-bromo-2-fluorobenzyl)piperidin-4-yl)-1*H*-benzo[*d*]imidazol-2(3*H*)-one) were meta (β-arrestin 2 E_max_ = 66%) to each other (Figure 2, Table 1b). Both compounds had similar G-protein potency and efficacy. Taking together comparative bias studies with biased/balanced agonist pairs, namely SR 17018 and SR11501 [51] and compounds **2** and **3** on this template, shows how small changes of chemical structure can lead to the engagement and disengagement of β-arrestin 2 while retaining G-protein potency. Additional studies are still ongoing, which the authors hope will guide the design of safer analgesics in the near future.

**Carfentanyl amides**

Compounds in the fentanyl class are believed to recruit β-arrestin 2 and are arrestin biased [33]. The introduction of a cycloheptyl amide substituent (*N*-cycloheptyl-1-phenethyl-4-(*N*-phenylpropionamido)piperidine-4-carboxamide, **MP102**) into the fentanyl moiety leads to MOR agonists which retain G-protein signaling but lose arrestin signaling. Interestingly, two analogs in the same series with a t-butyl amide group (*N*-(*tert*-butyl)-1-phenethyl-4-(*N*-phenylpropionamido)piperidine-4-carboxamide, **MP105**) and cyclopropyl amide group (*N*-cyclopropyl-1-phenethyl-4-(*N*-phenylpropionamido)piperidine-4-carboxamide, **MP103**) instead of cycloheptyl amide moiety showed higher efficacy in the β-arrestin 2 assay compared to **MP102,** again suggesting that small changes in structure of the analog lead to differential signaling (Table 1). A lead compound in this series **MP102** [25,52] exhibited moderately potent analgesia with significantly reduced respiratory depression, constipation, and physical dependence while showing analgesic tolerance and reward behavior. The role of DOR agonism in the actions of **MP102** may play a key role in vivo and needs to be investigated with future analog design.

**Controversy on biased agonists of the MOR**

The whole concept which states that G-protein biased agonists of the MOR that do not recruit β-arrestin could significantly improve the therapeutic window and are less prone to the development of classical opioids side effects still remains under very sensitive scrutiny. Indeed, several recent studies ask for very careful consideration of the in vitro/in vivo data, given that β-arrestin 2 signaling might not be directly or indirectly involved in opioid-induced respiratory depression, constipation, or withdrawal [27,53,54]. Among the most important findings, the respiration of morphine was found to be β-arrestin 2-independent. In addition, mice with mutations in the C-tail with a series of serine- and threonine-to-alanine mutations that is likely to lead to less recruitment of β-arrestin 2 still retained respiratory depression, constipation, and withdrawal of opioids. The results contrast with KO mice data from β-arrestin 2 in the S129/C57BL/6 mixed strain mice. Consistent with past results, tolerance was attenuated, and the analgesic duration of action was prolonged in these mutant mice. These controversial results mainly emphasize the critical need for novel pharmacological tools, which could help in probing the opioid-signaling system.

## 3. KOR Biased Agonism

**Figure 3:**
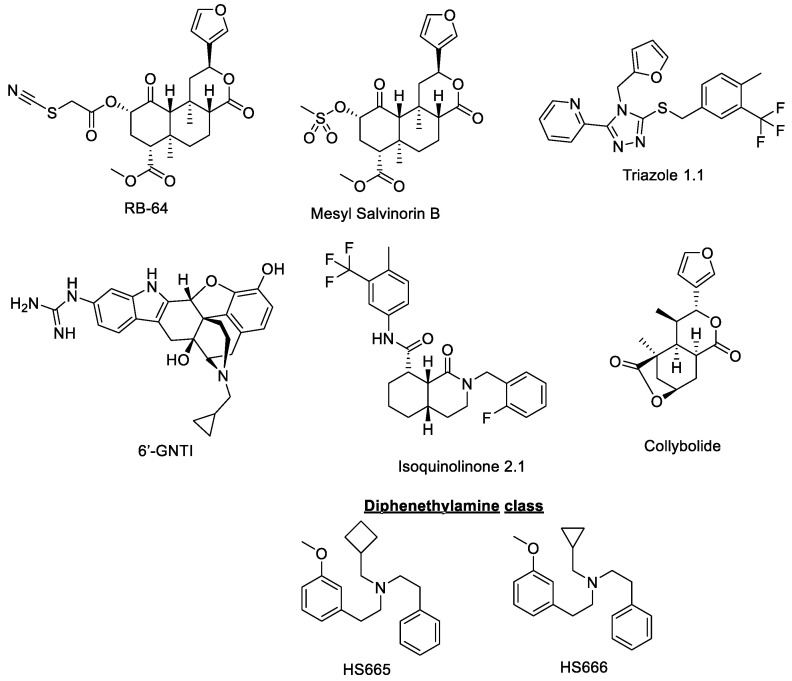
Structures of KOR biased ligands reported having different levels of β-arrestin 2 recruitment.

The ubiquitous distribution of κ-opioid receptors (KORs) across the peripheral and central nervous system and their involvement in a wide array of functions such as motor control, nociception, and consciousness have made KOR a promising target in the pain management field [55]. Interestingly, KOR agonists do not produce the common side effects associated with classical opioids such as respiratory depression and overdose, and do not activate the reward pathway [55]. However, the therapeutic utility of full KOR agonists is decreased by other effects including dysphoria, sedation, anxiety, and depression which have restricted the clinical development of such drugs. Nevertheless, recent studies proved that β-arrestin 2 recruitment and subsequent p38 phosphorylation is required in order to trigger aversion [56,57], whereas this is not the case for the analgesic effect. In addition, targeting KOR have been associated with reducing itch/pruritis as potential therapeutic action [58]. These findings suggest that functionally selective KOR agonists that are able to selectively activate G-protein signaling without activating p38α MAPK and thus causing side effects may have therapeutic potential as non-dysphoric antipruritic analgesics or could be used as adjuvants to potentiate MOR-targeting analgesics such as morphine. A list of ligands not recruiting β-arrestin 2 at KOR is shown in Figure 3. In this case U50,488H and Sal A are considered balanced agonist comparator drugs. A non-exhaustive list of KOR biased ligands with potency and efficacy at the G-protein and the β-arrestin2 pathways is shown in Table 2.

**RB64**

22-thiocyanatosalvinorin A, or RB-64, was first synthesized in the laboratory of Dr. Jordan Zjawiony as a semi synthetic structural derivative of the centrally active salvinorin A (extracted from the plant *Salvia divinorum*) compound [59]. This agent was described as a G-protein biased full agonist of the KOR with a bias towards G-protein signaling relative to salvinorin A by the Bryan Roth laboratory. In vitro studies showed indeed that RB-64 recruits β-arrestin 2 but acts primarily as a very potent agonist of the G-protein pathway. In the same study, the antinociceptive effect of RB-64 was tested in the hot plate assay along with U69593 and salvinorin A in wild-type, β-arrestin 2 KO, and KOR KO mice. RB-64 showed a significant and long-lasting analgesic effect in wild-type and β-arrestin 2 KO mice while no antinociception was detected in KOR KO mice, suggesting that this effect was essentially KOR-mediated.

Surprisingly, and in contrast to expectations, this agent (along with salvinorin A and U69593) produced significant aversion when it was tested in the CPP/CPA paradigm in both wild-type and β-arrestin 2 KO mice. Thus, these findings would suggest that G-protein pathway mediates KOR-induced dysphoria. Another feature of this study was that RB-64, even at a very high dose, did not impair motor coordination and the rotarod performances in wild-type and β-arrestin 2 KO mice contrarily to the two other unbiased standards. Given that salvinorin A and U69593 do recruit β-arrestin 2, this would mean an important role for β-arrestin 2 in the locomotor circuitry.

**Mesyl salvinorin B**

Similar to RB-64, ((2S,4aR,6aR,7R,9S,10aS,10bR)-9-(methanesulfonyloxy)-2-(3- furanyl)dodecahydro-6a-10b-dimethyl-4,10-dioxy-2H-naptho-[2,1-c]pyran-7-carboxylic acid methyl ester), or Mesyl salvinorin B, is a semi-synthetic structural derivative of Salvinorin A which was synthesized in the laboratory of Dr. Thomas E. Prinsinzano in order to improve Salvinorin A’s poor pharmacological profile [60]. Mesyl salvinorin B has a mesylate substitution at the C-2 position on its scaffold and has similar binding affinity for KOR compared to Salvinorin A (Mesyl Sal B K_i_ = 2.3 ± 0.1 nM and Sal A K_i_ = 1.9 ± 0.2 nM in CHO KOR) [60]. It has been shown to act as a G-biased full agonist of the KOR and induce both β-arrestin 2 recruitment and G-protein stimulation in vitro, the latter with a stronger potency. This agent has also been found to display a wide array of therapeutic effects such as the antinociception or attenuation of drug seeking behavior in rodents. Indeed, in the tail-withdrawal assay, Mesyl salvinorin B displayed antinociception but appeared to be a weaker analgesic with reduced potency (EC_50_ = 3.0 mg/kg, Sal A EC_50_ = 2.1 mg/kg) and efficacy (E_Max_ = 38%, Sal A E_Max_ = 87%) compared to its parent derivative Sal A. The same study also revealed a significant effect of this agent on cocaine-induced hyperactivity and seeking behavior. Mesyl salvinorin B did not negatively affect sucrose self-administration, which is a preclinical measure of anhedonia in male Sprague Dawley rats. Interestingly, this agent did also not induce aversion when tested for CPA and did not impair locomotor activity on the rotarod. Additionally, another study showed that Mesyl salvinorin B significantly reduced excessive alcohol drinking [67].

**Triazole 1.1**

[2-(4-(furan-2-ylmethyl)-5-((4-methyl-3-(trifluoromethyl)benzyl)thio)-4H-1,2,4-triazol-3-yl)pyridine], or triazole 1.1, is a compound that was first discovered in 2013 as the result of a collaboration between 5 different research institutes [65,68]. It was selected after high-throughput screening (HTS) of a huge library of KOR agonists because of its high degree of bias within the collection, its efficacy in the warm water tail immersion assay, and its ability to cross the blood–brain barrier efficiently when administered systemically. Triazole 1.1 is a full agonist of the KOR, which, compared to both U50,488H and U69,593, displayed remarkable G-biased agonism. This agent exhibited a potent antinociceptive effect in the warm water tail withdrawal assay that was comparable to that achieved with U50,488H in mice. In the same study, Norbinaltorphimine (NorBNI) was found to fully reverse the analgesia produced by triazole 1.1 and no effect was observed in KOR KO mice, which demonstrated that this effect was KOR mediated. KOR agonists are known for their potential antipruritic activity, which is why this compound was also tested in the mouse non-histamine pruritis model [58,69]. Triazole 1.1 was uncovered to significantly suppress chloroquine phosphate–induced scratching with an effect comparable to that of U50,488H. The antipruritic effects of triazole 1.1 were also blocked by a 24h pretreatment with NorBNI, which confirmed that the effect was KOR-specific. However, a very recent study on the effects of typical and atypical KOR agonists underlined that triazole 1.1 had no effect on its own, albeit reducing overall levels of scratching during time course determination [70]. In addition, an analysis of the locomotor activity showed that treatment with Triazole 1.1 did not affect locomotion at any of the doses tested, which was in contrast to what was observed with U50,488H. The same study also analyzed dopamine concentrations in the nucleus accumbens ((NAcc) which can be linked to both sedation and dysphoria in animals [61]. Contrary to the balanced agonist U50,488H, triazole 1.1 did not alter dopamine release in the brain nor affected significantly ICCS in rats at doses which induced in vivo analgesia.

**Diphenethylamines HS665 and HS666**

The first series of diphenethylamine derivatives was reported in 2012 by Drs. Helmut Schmidhammer and Mariana Spetea research groups as novel selective KOR ligands on the basis of previous work on the Dopamine D_2_ receptor agonist RU 24213 [71,72]. This agent displayed moderate activity on KOR and acted as an antagonist of the KOR. Further chemical derivatizations led to the identification of two leads, namely HS665 and HS666 (with an *N*-cyclobutylmethyl (N-CBM) and a *N*-cyclopropylmethyl (N-CPM) moiety, respectively), which exhibited great affinity and potency toward the KOR. More specifically, HS665 acted as a highly selective and full KOR agonist, while HS666 was a selective KOR partial agonist. Both these agents exhibited very weak partial agonism for β-arrestin 2 recruitment in contrast to U69,593 which robustly recruited β-arrestin 2 (using the DiscoveRx PathHunter β-arrestin 2 assay). In the warm water tail withdrawal assay, both compounds elicited dose-dependent potent analgesic effect characterized by a short onset in wild-type and MOR-KO C57BL/6J mice (WT ED_50_ = 3.74 (2.98–4.78) nmol and 6.02 (4.51–8.08) nmoL for HS665 and HS666, respectively). The same experiments were conducted in KOR-KO mice with no antinociception detected, which proved that the effects was KOR-specific. Noteworthy, these agents and the standard U69,593 were all administered intracerebroventricularly. The same study investigated the behavioral effects of HS665 and HS666 in vivo. Interestingly, neither of these agents significantly impacted motor performance at any time point and HS666 did not induce CPP/CPA (Conditioned Place Preference/Conditioned Place Aversion) while HS665 produced significant place aversion when administered 5 times their analgesic ED_90_ [62]. It is not clear if HS666 did not show CPA because it was a partial agonist or if this was because of its G-protein bias. The pharmacological profile of HS665 matched RB64. Another study from the same group focused on the development of novel derivatives based on the same structural scaffold and expanded the structure-activity relationships (SARs) of the original series [73]. In this study, HS665 and HS666 pharmacology was studied subcutaneously in CD1 mice. Both drugs showed analgesic action in acetic acid writhing assay while demonstrating no sedation or motor impairment. In addition, the introduction of bulkier *N*-substituents and additional hydroxyl groups resulted in the identification of novel, very potent (picomolar activity), and selective KOR ligands which displayed analgesia with a reduced liability profile, reflected by the lack of sedation and motor impairment. β-Arrestin 2 was not measured with any analogs in this series and CPA with lead compounds was also not evaluated. Similarly, the Kreek group evaluated additional N-substituents (N-cyclopentyl and N-cyclohexyl) and substituted the phenyl ring with a pyridine ring [74]. All analogs still retained the G-biased signaling seen with the parent HS666 on which these analogs were based upon. Detailed evaluations on these compounds are awaited.

**6′-GNTI**

Originally synthesized by Dr. Philip S. Portoghese and his research group in 2001, 6′-guanidinyl-17-(cyclopropylmethyl)-6,7-dehydro-4,5α-epoxy-3,14-dihydroxy-6,7-2′,3′-indolomorphinan dihydrochloride, more commonly known as 6′GNTI [75], is a derivative of the highly potent DOR-selective antagonist Naltrindole (NTI). It was initially proposed to act as a potent DOR-KOR heteromer selective ligand and was found to interact differently with the KOR and DOR owing to its guanidinium side chain in the 6′ position. Interestingly, a close derivative known as 5′-GNTI was found to be a selective antagonist of the KOR. Recent studies suggest 6′-GNTI is a KOR biased agonist with preferential activation of the G-protein over the β-arrestin 2 pathway [76]. More specifically, 6′-GNTI acts as a G-protein partial agonist of the KOR at low μM concentrations and does not stimulate β-arrestin 2 recruitment in the sub-mM range, acting as a β-arrestin 2 antagonist. These results were confirmed by another group that looked at drug-induced receptor internalization levels as an indirect measure of β-arrestin 2 recruitment [63]. While U50,488 and EKC led to robust receptor internalization, 6′-GNTI did not have a significant impact and potently inhibited EKC KOR-induced internalization. Additionally, 6′-GNTI was proven to be a potent analgesic in several assays and strains of rodents. It was found to produce potent analgesia in the radiant heat tail-flick assay in 129S6 and CD-1 mice and was also effective in completely blocking PGE_2_-induced thermal allodynia when administered to BK-pretreated hind paws in rats. Perhaps due to its lack of selectivity for the KOR, the analgesic effects were completely reversed when prior pretreatments with either NorBNI or Naltrindole (NTI) were administered in vivo.

Additional biological studies on the striatal neurons showed that the stimulation of the KOR by 6′-GNTI triggers the activation of the Akt pathway but not the phosphorylation of the ERK1/2 proteins, which is unusual when compared to a non-biased agonist such as bremacozine or U69,593. The same study also showed that in striatal neurons from β-arrestin 2 knockout mice, there was residual ERK stimulation by 6′-GNTI and that this phenomenon was β-arrestin 2-dependent [64]. In contrast, Akt phosphorylation was symptomatic of the G-protein pathway activation.

At last, a study from 2016 investigated the potential of 6′-GNTI as an anticonvulsant/antiseizure agent, which is another area of interest for the development of KOR agonists. The authors showed that (10–30 nmol) 6′-GNTI significantly reduced paroxysmal activity in the mouse model of intra-hippocampal injection of kainic acid (acute seizures), with an effect almost comparable to that of U50,488H. The effects were also completely reversed after administration of the selective KOR antagonist 5′-GNTI. They finally also confirmed that 6′-GNTI does not induce CPA nor influence motor activity, in contrast to U50,488H which displayed strong place avoidance [77].

**Isoquinolinone 2.1**

2-(2-Fluorobenzyl)-*N*-(4-methyl-3-(trifluoromethyl)phenyl)-1-oxo-octahydroisoquinoline-8- carboxamide, or Isoquinolinone 2.1, is an agent that was first published in 2013 as the result of a study led by two teams from the Scripps Research Institute and the University of Kansas [65]. Initially, the isoquinolinone scaffold emerged from a 72-member library prepared by a tandem Ugi reaction and Diels–Alder addition reaction and screening for binding at potential GPCR targets by the NIMH Psychoactive Drug Screening Program [78]. Further SARs and chemical optimizations through domino acylation/Diels–Alder addition led to the identification of Isoquinolinone 2.1 as a potential lead [79]. Like Salvinorin A, this agent lacks the basic nitrogen center common in small molecule KOR ligands scaffolds. Isoquinolinone 2.1 was reported to act as a potent and highly selective KOR agonist, biased toward the G-protein signaling pathway with minimal β-arrestin 2 recruitment. In their study, the authors assessed G-protein signaling and β-arrestin 2 recruitment in different cell lines (CHO-hKOR and U2OS-hKOR-β-arrestin 2-EFC or U2OS-hKOR- β-arrestin 2-GFP) and found this agent to be G-protein biased [65]. Downstream ERK1/2 phosphorylation was also investigated as an additional measure of β-arrestin 2 recruitment, with no visible recruitment detected. Of importance, ERK1/2 activation can be misleading in quantifying β-arrestin 2 recruitment given its involvement in various signaling pathways, which was judiciously pointed out by the authors.

Finally, the intraperitoneal administration of 30 mg/kg of this drug produced potent antinociceptive effect in the warm water tail-flick assay similar to that seen with the selective KOR agonist U50,488H, with the effects peaking at 20 min post drug treatment in C57BL/6J mice.

The study, however, did not include any side effects or behavior profiling, thus it is difficult to evaluate the safety profile of this compound compared to other KOR modulators.

**Collybolide**

Initially extracted from the mushroom *Collybia maculata* (*Basidomycota*) in 1974 by the group of Dr. Pierre Potier, Collybolide (Colly) is a natural product pertaining to the class of sesquiterpenes [80]. It was only later in 2016 that a study demonstrated the potential of Colly and its diastereoisomers (9-epi-Colly) in pain attenuation [66]. Due to its structural similarities with Salvinorin A (a common furyl-δ-lactone motif), this agent was tested on human hMOR, hDOR, and hKOR and was found to be very selective of KOR. When the authors looked at functional selectivity, they showed that Colly was a very potent agonist in [^35^S]GTPγS assays (EC_50_ ≈ 1 nM) and dose-dependently inhibited adenylyl cyclase activity, but was also potent for ERK1/2 phosphorylation suggesting that it acts as a biased agonist of the hKOR though β-arrestin 2 recruitment with this natural product remains unknown [66]. The effects were reversed upon treatment with NorBNI showing a KOR-mediated effect. Interestingly, the epimerization of Colly at C9 reduced the agonistic activity and signaling which would indicate that the C9 position is critical to the full binding and signaling of Colly on hKOR. More importantly, Colly was found to exhibit potent antinociceptive effect in the tail-flick assay but was also aversive in mice (when tested in the CPA paradigm), which is similar to what was observed with Salvinorin A. However, at the same doses of both Colly and Salvinorin A (2 mg/kg), only Colly was uncovered to significantly attenuate chloroquine-mediated scratching behavior, with the effects here again reversed upon the administration of NorBNI. Finally, the authors noticed that this agent could exhibit antidepressant and anxiogenic activity in the forced swim test and open field test instead of prodepressant and anxiolytic phenotype expected of classical KOR agonists [66].

## 4. Biased Agonism on DOR

The first unambiguous evidence for DOR action in antinociception came with the isolation of the classical DOR ligand deltorphin II (Tyr-D-Ala-Phe-Glu-Val-Val-Gly-NH2, naturally occurring and stable DOR peptide) with 0.8 nM potency in mouse vas deferens, first isolated from the skin of a Phyllomedusa species in 1989 by Erspamer and coworkers [81]. It proved to be 13 times more potent than the other selective DOR ligand DPDPE ([D-Pen2, D-Pen5] enkephalin) in a mouse tail-flick test upon intracerebroventricular administration with peak effect close to 10 min and antinociceptive duration of 40 to 60 min. It also displayed 15 times improved selectivity to DOR over MOR [81], making it an extremely useful tool in untangling DOR function.

Later investigations revealed that DOR agonists are poor analgesics in acute pain but they are effective in animal models of chronic inflammatory and neuropathic pain, and specifically alleviate persistent pain [82]. DOR agonists also show anti-allodynic and anti-hyperalgesic properties. There is also an inhibitory DOR tone, which reduces nociceptive responses under the conditions of persistent pain [83]. The lack of DOR receptors results in anxiogenic and depressive-like behavior [84], while DOR opioid receptor agonists produce anxiolytic and anti-depressant effects [85], demonstrating the importance of DOR in regulating emotional responses.

Under basal conditions, DOR is located predominantly intracellularly, but inflammation produces a dramatic change in DOR density leading to up-regulation and membrane targeting of the receptor [86]. Unlike MOR, DOR are predominantly targeted for degradation upon internalization [87].

Agonist activity on DOR does not lead to adverse effects associated with MOR agonists like respiratory depression, addiction, or constipation [88], but agonists were thought to display proconvulsive activity [89]. However, more recent research indicates that this adverse effect is ligand-specific and only SNC80 and AZD2327 evoke convulsions, hyperlocomotion, and receptor sequestration [90,91].

DOR activation reverses the decrease in TrkB protein expression after ischemia and reduces brain ischemic infarction while DOR inhibition aggravates the ischemic damage [92].

Unlike MOR and KOR, which have been heavily investigated for biased signaling, there is a paucity of ligands at DOR which exhibit G-protein bias. Also, in addition to β-arrestin 2, β-arrestin 1 also plays a key role in DOR mediated behaviors. This section will attempt to provide a mechanistic view of bias and evaluate the ligands which are known to display bias in vitro.

**DOR and biased signaling: mechanistic overview**

The high-resolution (1.8 Å) crystal structure of the human DOR in the inactive state was presented by Fenalti and coworkers in 2014 [93]. Similar to the NOP receptor structure, the ICL3 adopts a closed inactive state conformation stabilized with an H-bond network, unlike in many GPCR structures where the inactive state is stabilized by an “ionic lock”. The DOR sodium ion cavity is formed by 16 residues, 15 of which are conserved over class A GPCRs. A polar interaction network in the seven-transmembrane bundle core around the sodium ion stabilizes a reduced agonist affinity state modulating signal transduction. Disrupting this interaction network through mutagenesis transform classical DOR antagonists such as naltrindole into potent β-arrestin2 -biased agonists, possibly opening up new routes towards biased signaling [93]. Active-like state DOR structures in complex with a peptide (2.8 Å resolution) and a small-molecule agonist (3.3 Å resolution) obtained in 2019 revealed polar networks around the conserved D128^3.32^ residue with rearrangements in the agonist-bound binding pocket upon DOR activation [94]. This residue is crucial for receptor activation as opioid agonists that contain a basic nitrogen interacting with D^3.32^ extend deeper into the binding pocket compared to structurally similar antagonists. They also found changes in the nonconserved ECL3 during activation, which makes R291^ECL3^ available for binding pocket interactions, notably upon the binding of endogenous peptides. Unlike peptides, DOP-selective small molecules address the nonconserved extracellular ends of helices VI and VII with their *N*,*N*-diethylbenzamide moiety, which leads to their selectivity over MOP and KOP due to steric clashes in the same region of the latter receptors [94].

In the 1990s, there was growing evidence that DOR receptors are differentially desensitized by different agonists. Notably, in 1999, Allouche and co-workers noted that in SK-N-BE cells pre-challenged either with alkaloid or peptide agonist, cross-desensitization occurred that was less marked when cells were pretreated with peptide agonists and then challenged with etorphine than the other way around. Later developments showed that the DOR receptor arrestin-mediated internalization seems to be linked to the development of analgesic tolerance and low-internalizing agonists might have a decreased tendency to induce convulsions [85].

Bradbury and co-workers showed in HEK cells expressing high number of DOR that the degree of DOR Ser^363^ phosphorylation stimulated by different agonists and the ability of the agonist to induce internalization are related, while there was no correlation between G-protein activation and receptor phosphorylation/internalization [95].

Another study revealed that Ser^363^ in the δ-opioid receptor (DOR) determines the different abilities of the DOR agonists DPDPE and TIPP to activate ERK by G-protein- or β-arrestin-dependent pathways [96]. DPDPE employed G protein as the primary mediator to activate the ERK cascade in a Src-dependent manner, whereas TIPP mediated through the β-arrestin 1/2- mediated pathway. When Ser^363^ was mutated, DPDPE gained the ability to utilize β-arrestin 1/2 as scaffolds to assemble a complex with kinases of the ERK cascade accompanied by a decrease in the desensitization of ERK signaling, indicating that β-arrestin-dependent ERK activation might play a role in preventing DOR desensitization [96].

Qiu and co-workers showed on an all Ala mutant of DOR phosphorylation sites that DOR can undergo phosphorylation-independent receptor desensitization and internalization as well. Without phosphorylation, agonist-activated DOR interacted with β-arrestin 1 and β-arrestin 2 similarly, whereas phosphorylation promoted the receptor selectivity for β-arrestin 2 over β-arrestin 1, presumably through the phosphorylated Thr/Ser residues of the carboxyl tail of DOR. The all Ala mutant displayed no interaction between β-arrestins and the carboxyl tail [97].

Aguila demonstrated that the human DOR is differentially regulated via β-arrestin 1-biased mechanisms depending on the ligand [98]. Namely, the reduction of the endogenous level of β-arrestin 1 in SK-N-BE cells only diminished peptide-induced (DPDPE and deltorphin I) hDOR desensitization, while etorphine induced desensitization remained at the same level. However, when examining endocytosis, β-arrestin 1 depletion led exactly to the opposite effect, diminishing only etorphine induced endocytosis. Given that DOR binds β-arrestin via two distinct domains [99], it is possible that the two regions would be differentially unmasked upon binding of peptidic or alkaloid ligands at the receptor, either leading to receptor uncoupling (in case of peptides) or to receptor endocytosis (in case of etorphine) [98].

Pradhan and coworkers showed DOR receptors are in a pre-engaged complex with β-arrestin 2 at the cell membrane [100]. High-internalizing agonists like SNC80 seem to induce receptor phosphorylation, preferentially recruit β-arrestin 1, and result in receptor internalization and degradation. Low-internalizing agonists like ARM390 and JNJ20788560 rather strengthen the engagement between DOR and β-arrestin 2, protecting against acute behavioral tolerance.

They also reported that the anti-allodynic effects of the high-internalizing agonist SNC80 were modulated by β-arrestin 1 and not β-arrestin 2. KO of β-arrestin 1 resulted in increased drug potency, duration of action, and decreased acute tolerance. No change in the antihyperalgesic effect of SNC80 was observed in β-arrestin 2 KOs. In contrast, β-arrestin 2 KO resulted in a gain of acute tolerance to low-internalizing agonists, suggesting that β-arrestin 2 enhances delta opioid receptor resensitization.

In the same study, live-cell imaging revealed that there is a basal engagement between DOR and β-arrestin 2 at the cell membrane, an interaction not observed with β-arrestin 1. The β-arrestin 2 interaction was strengthened with ARM390, while binding of a high-internalizing agonist produces preferential interaction between the receptor and β-arrestin 1 [100].

Similarly, Vicente-Sanchez found that β-arrestin 1 mediates the development of tolerance to the antihyperalgesic and convulsive effects of SNC80, but not tolerance to the antihyperalgesic effects of ARM390 and that DOR remained functionally coupled to G-proteins in β-arrestin 1 KO mice chronically treated with SNC80 [101].

In 2018, Dripps and coworkers showed [102] that DOR-induced convulsions are mediated with different signaling mechanisms than antihyperalgesia and antidepressant-like effects, notably that G_αo_, but not arrestins play a role in regulating the acute antihyperalgesic and antidepressant-like effects while β-arrestin 1 negatively regulates DOR-mediated convulsions, G_αo_ not playing a function in this respect. Similarly, the loss of RGS4 potentiated the antinociceptive, antihyperalgesic, and antidepressant effects of SNC 80 likely due to prolongation of DOR-mediated G protein signaling, while it did not affect convulsions. SNC80-induced convulsions were unaffected in β-arrestin 2 knockout mice but potentiated in β-arrestin 1 knockout mice [102].

**Figure 4:**
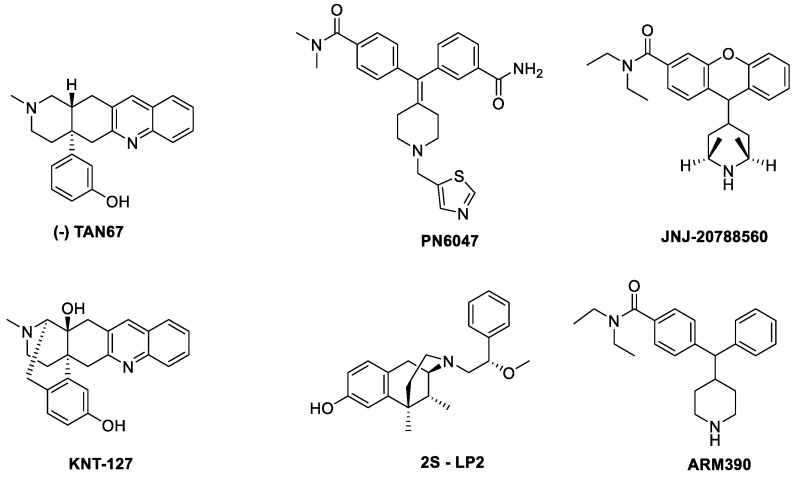
Structures of DOR biased ligands reported having different levels of β-arrestin 2 recruitment.

Altogether, it seems there are two distinct types of DOR agonists, one, like SNC80 leading to convulsions, hyperlocomotion, and receptor sequestration, ultimately leading to tolerance to the analgesic as well as to locomotor effects. The other type of agonists, which constitute the majority, does not lead to sequestration and only leads to tolerance to the analgesic effects upon chronic treatment [90]. A part of these effects but not all could be linked to variable efficacy for arrestin-receptor interactions [103]. Ultimately, while receptor sequestration shuts down signaling at the plasma membrane, it might open up new therapeutic opportunities. A recent article by Jimenez-Vargas showed that SNC80 and DADLE ([D-Ala^2^, D-Leu^5^]-Enkephalin), both of which strongly internalize DOR, activate G_αi/o_ in endosomes and recruit β-arrestin 1/2 both to the plasma membrane and endosomes [104]. Furthermore, nanoparticle-encapsulated agonists (DADLE) target endosomal DOR and provide long-lasting antinociception through the long-lasting inhibition of mechanically evoked activation of colonic nociceptors, providing evidence that DOR in endosomes might be a superior therapeutic target for inflammatory pain [104]. The structures of DOR ligands are shown in Figure 4, while data available in the literature is summarized in Table 3.

**TAN67**

TAN67 was designed in 1998 by Nagase and coworkers as an enantiomeric mixture based on the “message-address” concept. With a high affinity (K_i_ = 1.12 nM) and potency (IC_50_ = 6.61 nM in mouse vas deferens), it shows 2070-and 1600-fold selectivity on DOR over MOR and KOR, respectively. When administered subcutaneously, it produces an inhibition of the acetic acid induced abdominal constriction response. Later, Nagase and coworkers showed that the antinociceptive effects originate from the (-) isomer [108].

TAN67 seems to have similar potency as Leu-enkephalin both in the cAMP (around 3 nM) and β arrestin-2 recruitment assays (10–30 nM range) but has a significantly lower efficacy (41% compared to Leu-enkephalin) in the β-arrestin 2 recruitment assay [107]. As DOR agonists, both compounds alleviate alcohol withdrawal induced anxiety in mice, but only TAN67 reduced alcohol consumption [109], which might be a consequence of its diminished ability to recruit β-arrestin 2 [107].

**PN6047**

PN6047 is an orally bioavailable, DOR-selective G-protein biased agonist with potent antihyperalgesic efficacy in preclinical models of chronic pain. PN6047 elicited a maximal response in the BRET G-protein activation assay equivalent to that of SNC80, but with 10-fold greater potency. PN6047 is significantly biased toward G-protein activation over β-arrestin recruitment, being a particularly weak recruiter of β-arrestin 1, relative to SNC80.

In line with its G-protein efficacy, PN6047 elicited ERK1/2 activation equivalent to that of SNC80, but as a result of its limited ability to recruit arrestins, it is a partial agonist with respect to internalization.

PN6047 does not appear to have proconvulsive activity or induce analgesic tolerance. Unlike SNC80, repeated administration of PN6047 does not induce analgesic tolerance over a 16-day dosing regimen, maybe as a consequence of its limited ability to induce internalization. Like other DOR agonists, the action of PN6047 is selective for chronic pain states. In the forced swim test, PN6047 decreased immobility, consistent with the antidepressant-like effects of DOR agonists [105].

**KNT127**

KNT-127 displays high in vitro affinity for DOR (Ki = 0.16 nM) [110] with a potency of 2.0 nM in the cAMP assay, 3.3 nM in β-arrestin 2 recruitment assay, but with a diminished efficacy when compared to DPDPE (6.3 nM in cAMP, 12.6 nM in β-arrestin 2 recruitment assay)[107], and low affinity for MOR and KOR receptors (Ki = 21.3 and 153 nM) [110]. Its administration leads to a strong analgesia in mouse chemical pain assays [111] and significant antidepressant effects, while not causing convulsion, locomotor activation, amnesia, or coordination deficits [112].

KNT-127 (5 mg/kg) fully reversed both thermal hyperalgesia and mechanical allodynia at first administration, and this effect gradually diminished over 5 days, and tolerance to the analgesic effects of KNT-127 develops independently from pain modality and mouse strain. Chronic KNT-127 induces in vivo tolerance to DOR receptor analgesia. Altogether, the KNT-127 profile is similar to that of other agonists like AR-M1000390, ADL5747, and ADL5857 [90].

**JNJ 20788560**

JNJ 20788560 has an affinity of 2.0 nM for DOR (rat brain cortex binding assay) and a potency of 7.6 mg/kg p.o. in a rat zymosan radiant heat test and of 13.5 mg/kg p.o. in a rat Complete Freund’s adjuvant RH test while being inactive in an uninflamed radiant heat test. Similar to ARM290, it does not recruit β-arrestin 1 but strengthens the receptor β-arrestin 2 interaction [100]. JNJ-20788560 does not produce gastrointestinal (GI) erosion, neither does it lead to the slowing of GI transit. JNJ-20788560 does not exhibit side effects like respiratory depression, withdrawal signs, self-administration behavior, muscular rigidity, or the development of tolerance [88].

**2*S*-LP2**

2*S*-LP2 is a biased agonist at MOR and mainly at DOR, showing a significant improvement over the *R* isomer of this compound.

During BRET studies in SH-SY5Y cell membranes DADLE promoted MOR/G-protein interaction with a potency of 6.89 and maximal effect of 81%. 2S-LP2 mimicked the maximal effect of DADLE (pEC_50_ = 6.89) but was 30 times more potent (pEC_50_ = 8.33, respectively). DADLE stimulated the interaction of the MOR with β-arrestin 2 with pEC_50_ of 5.86 and maximal effect of 57%. 2S-LP2 mimicked the stimulatory response of DADLE with slightly lower efficacy but 9 times higher potency. 2*S*-LP2 displayed a modest (<10 times) bias toward G-protein.

On DOR, DADLE displayed a pEC_50_ value of 7.23 and maximal effect of 42%. 2*S*-LP2 behaved similarly to DADLE. DADLE stimulated the interaction of the DOR with β-arrestin 2 with pEC_50_ of 7.69 and maximal effect of 47%. 2*S*-LP2 mimicked the maximal effects of DADLE, however, being less potent. 2*S*-LP2 showed a statistically significant and large (200-times) bias toward G-protein.

A robust antinociceptive effect was achieved at very low doses of 2*S*-LP2, with an ED_50_ of 0.6 mg/kg i.p. that revealed its highest effect already at 30 min post- administration. 2S-LP2 did not significantly affect behavior responses (i.e., locomotor activity and sedation) [106].

**ARM390**

**ARM390** is a close analogue of prototypical DOR agonist SNC80. **ARM390** in the neuroblastoma cell line SK-N-BE expressing only human DOR receptors experiments showed a weak affinity (K_i_ = 106 ± 34 nM) and potency (EC_50_ = 111 ± 31 nM) in cAMP assay. Like SNC80, ARM390 reduces CFA induced inflammatory pain, but unlike SNC80, it retains analgesic response for subsequent agonist injection [113] and does not cause convulsions or motor coordination deficits [90] Exposure to maximal inhibitory concentration of ARM390 leads to a rapid and strong DOR desensitization caused by uncoupling, as opposed to DPDE, Deltorphin I, and SNC-80 which desensitize through internalization [114].

ARM390 being a low-internalizing DOR opioid agonist has been suggested to be a consequence of its limited β-arrestin recruitment [100]. ARM390, while being a full agonist, exhibits lower potency than the other agonists and is significantly less potent than SNC80 in the β-arrestin recruitment assays.

## 5. Biased Agonism on NOP Receptor

In 1994, multiples groups reported a fourth member of the opioid receptor family that did not bind any natural or synthetic opioid ligands. Its natural ligand, N/OFQ, was first isolated the following year from hypothalamic tissue using a reverse pharmacology approach by Civelli in the CNS Department of Hoffmann-La Roche in Basel, Switzerland, and by the groups of Jean-Claude Meunier from the University of Toulouse, France, and Gilbert Vassart from the University of Brussels, Belgium.

Unlike the other natural opioid peptides that start with the canonical sequence YGGF (Tyr-Gly-Gly- Phe) at the N terminus (message domain of the peptide), N/OFQ sequence starts with FGGF (Phe-Gly-Gly-Phe).

NOP receptor is a Class A GPCR having similar intracellular coupling mechanisms to opioid receptors. N/OFQ produces anti-opioid hyperalgesic effects in supraspinal pain pathways, but analgesic effects in spinal pain pathways [115].

NOP is coupled to the inhibition of cAMP, activation of MAPK, activation of K^+^ conductance, inhibition of Ca^2+^ conductance, and the inhibition of neurotransmitter release like GABA, dopamine, and acetylcholine [115].

Unlike classical opioids, NOP receptor agonists affect nociceptive transmission in a site-specific and agonist-dependent manner, but effects also depend on the species tested, and the pain state of the animal [116].

NOP receptors and N/OFQ play an active role in pain transmission, and a mixed NOP receptor/MOR agonist is now in clinical trials, and selective NOP receptor agonists are examined as possible analgesics, although they are often very sedative [117]. Notable exemptions are cebranopadol and AT-121, perhaps due to ligand bias, although we were unable to locate a bias factor for the latter in the literature [118,119]. NOP receptor agonists are more effective in blocking chronic than acute pain for unknown reasons.

The activation of the N/OFQ-NOP system leads to anxiolysis [115,120] while its blockade produces antidepressant-like effects, and this effect can be reversed by NOP receptor agonists [121]. It has been suggested that antidepressant effects of NOP receptor antagonists are linked to restoring hippocampal neurogenesis by counteracting the inhibitory effects of the endogenous N/OFQ on monoaminergic systems and increasing the expression of neuronal factors such as FGF-2 [122].

Asth and coworkers showed that NOP receptor ligands are able to promote NOP receptor /β-arrestin 2 interaction are also able to induce anxiolytic-like effects in EPM (elevated plus maze), but compounds that inhibited the NOP receptor /β-arrestin 2 interaction produced antidepressant-like effects in the FST (forced swim test) in mice [123]. This implies that the action of NOP receptor ligands on emotional states is better predicted based on their β-arrestin 2 rather than G-protein efficacy. It is possible that NOP receptor antagonists also induce antidepressant effects by blocking the recruitment of β-arrestin 2 [123].

N/OFQ blocks opioid induced supraspinal analgesia and morphine reward in the CPP paradigm [124] and reduces morphine induced Dopamine (DA) release in the nucleus accumbens of conscious rats [125].

Treatment with selective NOP receptor antagonists prevented the development of tolerance following chronic treatment with morphine. Acute treatment with N/OFQ was unable to prevent the intravenous self-infusion rate of heroin ([115] and references therein).

N/OFQ attenuates the reinforcing and motivating effects of ethanol, perhaps due to its ability to alleviate negative affective states. N/OFQ prevents the expression of somatic and affective alcohol withdrawal in ethanol dependent rats. However, 3 weeks post intoxication, N/OFQ gave rise to anxiogenic like actions in ethanol dependent rats, while continuing to exert anxiolytic like effects in non-dependent control ([115] and references therein). It should be noted that similarly to NOP receptor agonism, NOP receptor blockade reduced alcohol drinking and seeking in laboratory animals and in humans. Thus, it has been proposed that the beneficial effect of NOP receptor agonists may depend upon rapid desensitization of the N/OFQ-NOP receptor system following administration [126].

**Mechanism of biased signaling on NOP receptor**

NOP receptors functionally recruit both β-arrestin 1 and β-arrestin 2, the kinetics of the recruitment being ligand specific [127]. There is evidence that β-arrestin 2 is involved in NOP receptor internalization processes [128]. The endocytotic activity of NOP receptor agonists is associated with their ability to induce receptor phosphorylation at Ser^346^, Ser^351^, and Thr^362^/Ser^363^; a direct positive linear correlation was observed between the phosphorylation at Thr^362^/Ser^363^ and receptor internalization as well as between phosphorylation at Thr^362^/Ser^363^ and GIRK channel activation. This phosphorylation pattern in the C-terminal domain proved to be agonist selective [129].

Besides the agonist and cell type, tissue environment might have a significant impact on NOP receptor internalization and arrestin recruitment properties [130]. In most cases, NOP receptor internalization starts rapidly with robust internalization at 1h post treatment in transfected cells [128]. Following endocytosis, NOP receptor may be targeted to either recycling endosomes for return to the cell surface or lysosomes/proteosomes for proteolytic degradation and downregulation [131].

Chang and coworkers were the first to identify biased signaling at NOP receptor. While they identified G-protein bias for multiple compounds, no arrestin biased compounds could be identified, which might suggest that arrestin recruitment to the receptor is dependent of G-protein activation, and may be consequential and conformationally additive to the activated receptor/G protein complex [127].

A more systematic study on bias of NOP receptor agonists was performed by Malfacini and coworkers in 2015 using bioluminescence resonance energy transfer (BRET) technology to measure the interactions of the NOP receptor with either G-proteins or β-arrestin 2. In contrast to previous studies on the constitutive activity of MOR [132], NOP receptor did not display spontaneous coupling between the NOP receptor and G-proteins. Malfacini and coworkers showed that NOP receptor internalization requires a clathrin-dependent endocytosis mechanism that is mediated by arrestins. Most NOP receptor agonists tested show a bias for the G-protein-mediated signaling interactions, and partial agonists on the G-pathway behaved as pure competitive antagonists of receptor/arrestin interactions [130].

**Figure 5:**
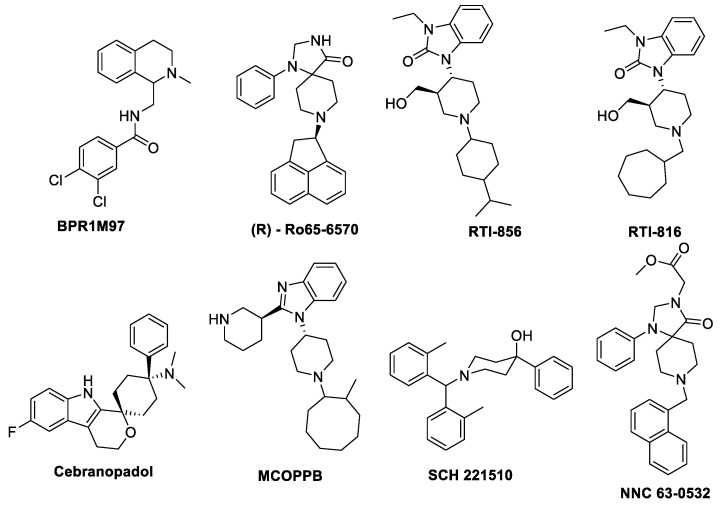
Structures of NOP receptor biased ligands reported having different levels of β-arrestin 2 recruitment.

Due to the lack of arrestin biased signaling, the relative role of G-protein and arrestin in mediating different actions is not completely understood. The structures of biased ligands at NOP receptor are shown in Figure 5, while data available in the literature is summarized in Table 4.

**BPR1M97**

BPR1M97 was first identified at the National Health Research Institutes of Taiwan and proved to be a potent MOR agonist and KOR agonist with moderate activity [135]. Later, the same group showed that it behaves as a balanced full agonist in cell-based MOR assays, similar in potency and maximal efficacy to dermorphine, but as a G-protein-biased full agonist of NOP receptor having slightly lower potency at decreasing the cAMP level than that of N/OFQ, while having a similar E_Max_ in HEK cells. Thus, it behaves as a dual NOP receptor/MOR agonist with 3-fold higher potency on the MOR system. BPR1M97 failed to trigger β-arrestin 2 recruitment altogether in CHO–NOP receptor. BPR1M97 exerts faster thermal antinociceptive effects at 10 min after subcutaneous injection and shows superior antinociceptive effect of mechanical and cold allodynia (acute pain) in cancer-induced pain than morphine, while causing less respiratory, cardiovascular, and gastrointestinal dysfunction at equi-antinociceptive doses. Notably, BPR1M97-treated mice recovered respiratory frequency at 30 min post-injection as opposed to morphine, where decreasing respiratory frequency could be observed until 60 min. In addition, BPR1M97 decreased global locomotor activity as compared with morphine, and induced less withdrawal jumping precipitated by naloxone, and showed lower cross tolerance in morphine-tolerant mice than morphine in BPR1M97-tolerant mice [134]. It is difficult to assess if NOP receptor bias plays a role in the in vivo actions of this drug, but most likely the polypharmacology with actions at NOP receptor and opioid receptors mediate these effects.

**Ro 65-6570**

Ro 65-6570 was reported by Wichmann in 1999, and proved to have a 10- to100-fold increased affinity towards NOP receptor as opposed to the opioid receptors as expressed by the pKi values (9.6 for NOP receptor and 8.4, 7.7, and 7.0 for MOR, KOR, and DOR receptors, respectively) from competitive binding experiments with [^3^H]-orphanin for N/OFQ in HEK293 cells, [^3^H]-naloxone (MOR, KOR), and [^3^H]-deltorphin (DOR) in BHK cell membranes [136]. In a BRET assay on HEK293 cell membranes, Ro 65-6570 exhibited a maximal effect not significantly different from that of N/OFQ, but was 5 fold less potent in the G-protein pathway [130]. In the NOP receptor /β-arrestin 2 assay, Ro 65-6570 showed a 50-fold loss of potency compared to N/OFQ. Comparing the ligand efficacy at G protein and β-arrestin 2 suggested that Ro 65-6570 behaves as a G-protein biased agonist.

Spontaneous locomotion in the elevation maze plus test and force motor performance were not significantly affected by Ro 65-6570 treatment [137,138]. Ro 65-6570 does not induce place preference, but co-administration (i.e., both compounds administered directly before the conditioning trial) reduced acquisition of condition place preference induced by opioids, but not by psychostimulants. Reduction of the rewarding effect of tilidine and oxycodone by Ro 65-6570 was reversed by the NOP receptor antagonist J-113397 [139].

**SCH221510**

SCH221510 displays at least 217-fold binding selectivity and 57-fold functional selectivity for the NOP receptor site, compared with the other opioid receptors, having a binding affinity of 0.3 nM in CHO cells which is 15-fold lower than the affinity of N/OFQ (0.02 nM), and has a functional in vitro potency (EC_50_) of 12 nM as measured by [^35^S] GTPγS binding to CHO cell membranes expressing NOP receptor. In the BRET assays in HEK293 cells, on the other hand, SCH-221510 displayed similar maximal effects but a 2-fold lower potency compared to N/OFQ in the G-protein assay, while it was able to promote NOP receptor/ β-arrestin 2 interactions with 10-fold less potency than N/OFQ. It displayed an almost 6-fold bias for the G-protein activation [130]. In preclinical animal models, SCH221510 produces robust and broad ranging anxiolytic-like effects in rat, gerbil, and guinea pig, that are similar to the effects produced by the benzodiazepine CDP, and do not decrease after a chronic dosing regimen. It produces anxiolytic-like activity at doses that do not produce nonspecific disruption of locomotor activity [140].

**Cebranopadol**

Cebranopadol behaves as a G-protein biased agonist at MOR where it recruits β-arrestin 2 with a 20-fold lower potency than for the activation of the G-protein pathway and particularly at NOP receptor, where it does not recruit β-arrestin 2. Meanwhile, its potency at MOR is 15-fold greater than at NOP receptors (0.18 nM and 3.24 nM, respectively, as compared to 6.92 nM for OFQ/N and 3.02 nM for dermorphin in the same BRET assay in HEK 293 cells). In vivo, cebranopadol exhibits highly potent and extremely long-lasting antinociceptive effects originating from both MOR and NOP receptors displaying higher analgesic potency against inflammatory than nociceptive pain, without eliciting sedation. The effects of cebranopadol in the tail withdrawal assay were sensitive to both SB-612111 and naloxone [133].

Cebranopadol, despite being a potent MOR agonist, produces only little opioid-type physical dependence in mice and rats, potentially due to its NOP receptor agonistic effects [141]. Linz and coworkers proved in 2017 that cebranopadol limits the respiratory depressant effect of its μ-opioid receptor agonist activity in rats is due to its NOP receptor agonist activity [118]. In some ways, the effects are similar to buprenorphine which shows a ceiling effect in respiratory depression with its NOP receptor actions being responsible for its particular pharmacological profile. Cebranopadol exerts potent antihyperalgesic, antiallodynic, and antinociceptive effects after local/peripheral, spinal, and supraspinal administration. After central administration of cebranopadol, antihyperalgesic efficacy is reached at doses that are not yet antinociceptive [142]. Cebranopadol was also shown to not induce either phosphorylation, or NOP receptor internalization [129].

**MCOPPB**

NOP receptor selective ligand MCOPPB is a G-protein biased full agonist, approximately 10-fold more potent than nociceptin and markedly less potent in arrestin recruitment displaying ∼ 10^5^-fold decrease in potency for arrestin coupling compared with G protein activation. MCOPPB shows a concentration-dependent or potency bias, as the ligand is a full agonist in both signaling pathways but distinguishes itself in its potency at G-protein versus arrestin signaling [127]. MCOPPB is a very potent agonist to activate the NOP receptor GIRK channels with an EC_50_ of 0.06 nM compared to N/OFQ (EC_50_ of 1.5 nM) for GIRK activation [129]. MCOPPB has an anxiolytic activity comparable to that of the benzodiazepine diazepam, but did not affect motor activity or memory function nor did it interact with alcohol at an anxiolytic dose in mice [143].

**NNC 63-0532**

NNC 63-0532 was reported by Thomsen in 2000 [144], and it shows moderate to high activity (70% inhibition in the cAMP assay) for MOR and KOR and for DOR and D_2_, D_3_ and D_4_ receptors. NNC 63-0532 showed about 20-fold selectivity for NOP receptor in radioligand binding assays over MOR and KOR (respective Ki values were 7.3 nM, 140 nM and 405 nM) and 14-fold when measuring displacement of radioligand binding to D_2_, D_3_ and D_4_ receptors (respective Ki values were 209 nM, 133 nM and 107 nM)

Besides its a NOP receptor selectivity, its arrestin recruitment could not be measured in HEK cells using BRET, which makes it a G protein-biased agonist exhibiting partial agonist activity with an efficacy of 71% and relatively low potency as indicated by a 130 fold shift in the value of EC_50_ in comparison with N/OFQ in cAMP inhibition assay in HEK cells [127]. NNC 63-0532 was not able to induce multisite phosphorylation of the NOP receptor [129].

**RTI–819 and RTI–856**

Both compounds show high selectivity for NOP receptor over all other opioid receptors (lowest ~30 fold on MOR for RTI–819 and ~100 fold for KOR for RTI–856)

Both RTI-819 and RTI-856 are partial agonists exhibiting similar efficacies (both around 75%) with respect to N/OFQ, but have a ∼10-fold difference in potency in the G-protein pathway (72.4 nM and 7.24 nM respectively as compared to 0.20 nM for N/OFQ), while they only very weakly induced βarrestin 1 and βarrestin-2 recruitment. In the same fashion as all other G-protein partial agonists, they show bias towards G-protein signaling owing to their very weak recruitment of arrestins. This might be an indication of arrestin not being recruited at detectable levels until a threshold level of G-protein receptor activation/saturation and G-protein-coupled receptor kinase phosphorylation is reached [127].

## 6. Future Directions and Conclusions

Ligand bias at opioid receptors has come a long way, yet many questions remain unanswered. At MOR, the response is mixed with attenuation of respiratory depression not correlating with βarrrestin-2 recruitment. The low respiratory depression of mitragynine(s) discussed in this manuscript may be a result of other targets, like DOR antagonism. Newer reports are emerging which suggest that low intrinsic efficacy maybe responsible for the lower respiratory depression of PZM21 and SR17108 [26] compared to fentanyl. Similarly, a possible reason why ligands appear as biased maybe due to the use of highly amplified systems where a partial agonist appears like a full agonist. Going forward, a potency biased model where a ligand shows an E_Max_ > 70% in both G and arrestin is proposed instead of an efficacy biased model where a ligand with <20% E_Max_ in the arrestin pathway ligand is characterized as biased [145]. It should be noted that the attenuation of side effects in β-arrestin 2 KO mice was only seen with morphine but not with methadone, oxycodone, or fentanyl, suggesting that how the ligand stabilizes the receptor may be more important and additional signaling circuits cannot be ruled out [146].

There is a desperate need for arrestin biased ligands at all opioid subtypes (MOR, KOR, DOR, and NOP receptor) to truly understand the pharmacology of the arrestin pathway with ligands. To the best of our knowledge, only fentanyl is arrestin biased at MOR. No such ligands exist at KOR, DOR, or NOP receptor. In a mice behavioral assay an arrestin biased agonist will ideally have a phenotype opposite to that of a G-biased agonist. For example, a MOR arrestin biased ligand should show lower analgesic efficacy and higher tolerance compared to a G-biased MOR agonist. Similarly, the propensity to cause convulsions for a DOR arrestin biased ligand will be higher over a G-biased agonist and a KOR arrestin biased agonist will have more sedation. At the neuronal as well as the cellular level, these arrestin biased drugs should lead to greater internalization of the receptor.

The synthesis and pharmacology of PR6047 suggests that bias at DOR may still hold potential in investigating functional selectivity and dissociating receptor induced adverse effects from its analgesia. Newer biased ligands with high selectivity for NOP receptor over MOR are required to correlate the in vivo pharmacology with ligand bias in the NOP receptor class.

A rational drug design of biased ligands is still lacking. Some correlations of bias can be drawn out of the MP1104-KOR structure where mutation of Y312W led to transformation of balanced agonist IBNtxA into a biased agonist at KOR [147]. The stabilization of the amide carbonyl group of IBNtxA [148] through H-bonding with the phenol of ‘Y’ holds the iodophenyl amide arm of the ligand in the TM2-TM3 region of KOR. Mutation of ‘Y’ to ‘W’ leads to loss of this interaction and flips the amide arm towards TM5-ECL2. The investigators hypothesized that ligands binding in this TM5-ECL2 region may lead to biased agonism, while ligands orienting towards TM2-TM3 may lead to balanced agonism. The same ligand IBNtxA at MOR was a biased agonist because the aminophenyl arm was oriented towards TM5-ECL2 region. Older studies from DOR inactive state structures (D95A, N131A) as well as KOR active state structures (N141A) [147] suggest that mutations in the Na^+^ binding pocket flip function of DOR antagonist as well non-selective opioid antagonists naloxone and nalterexone to β-arrestin 2 biased ligands suggesting another subpocket which may control arrestin engagement [93]. Not surprisingly, MOR variants in the C-tail of MOR also controls arrestin recruitment and control tolerance/dependence in vivo in mice as shown in elegant studies by Pan and co-workers at MSKCC [149]. The structures of biased ligands at opioids are presently missing and it is hoped that such structures of such biased ligands will greatly aid in structure-based design of biased opioids. Together, these studies are essential to identify receptor hot spots that lead to arrestin engagement and disengagement and to understand functional selectivity better.

## Figures and Tables

**Table 1:** Ligands targeting the MOR.

Ligand	Functional	G-Protein	E_max_	β-Arrestin2	E_max_	PubChem	Ref
	Selectivity	EC_50_ (nM)		EC_50_ (nM)		ID	
TRV130	G-protein	8.1 (cAMP)	84	7.3 (PathHunter)	15	66553195	[23]
Morphine	Balanced	7.4 (cAMP)	100	6.3 (PathHunter)	100	5288826	[23]
	agonist						
TRV130	G-protein	7.97 (Glosensor)	75	8.02 (BRET)	26	66553195	[24]
DAMGO	Balanced	8.58 (Glosensor)	100	8.3 (BRET)	100	5462471	[24]
	agonist						
TRV130	G-protein	7.9 (cAMP)	86	Inactive (PathHunter)	NQ	66553195	[25]
DAMGO	Balanced	8.4 (cAMP)	100	6.7 (PathHunter)	100	5462471	[25]
	agonist						
TRV130	G-protein	8.66 (cAMP)	86	7.71 (BRET)	58	66553195	[26]
	partial agonist						
DAMGO	Balanced	8.48 (cAMP)	100	7.55 (BRET)	100	5462471	[26]
	agonist						
PZM21	G-protein	7.73 (Glosensor)	83	7.68 (BRET)	32	121596705	[24]
DAMGO	Balanced	8.58 (Glosensor)	100	8.3 (BRET)	100	5462471	[24]
	agonist						
PZM21	G-protein	110 (BRET)	39	450 (BRET)	18	121596705	[27]
DAMGO	Balanced	390 (BRET)	100	1200 (BRET)	100	5462471	[27]
	agonist						
PZM21	G-protein	8.64 (cAMP)	84	7.56 (BRET)	59	121596705	[26]
	partial agonist						
DAMGO	Balanced	8.48 (cAMP)	100	1200 (BRET)	100	5462471	[26]
	agonist						
7-OH	G-protein	34.5 (BRET)	47	Inactive (BRET)	NQ	44301524	[28]
DAMGO	Balanced	1 (BRET)	100	NA (BRET)	100	5462471	[28]
	agonist						
7-OH	G-protein	53 (GTPγS)	77	Inactive (PathHunter)	NQ	44301524	[29]
DAMGO	Balanced	19 (GTPγS)	100	106 (PathHunter)	100	5462471	[29]
	agonist						
7OH	G-protein	7.8 (cAMP)	84	Inactive (PathHunter)	NQ	44301524	[25]
DAMGO	Balanced	8.4 (cAMP)	100	6.7 (PathHunter )	100	5462471	[25]
	agonist						
	Selectivity	EC_50_ (nM)		EC_50_ (nM)		ID	
Mitragynine	G-protein	1.7 (CHO)	82	Inactive	NQ	44301701	[29]
pseudoindoxyl				PathHunter			
DAMGO	Balanced	19 (GTPγS)	100	106 (PathHunter)	100	5462471	[29]
	agonist						
Herkinorin	G-protein	500 (CHO)	130	No internalization of	NQ	11431898	[30]
				βarr2-GFP			
Herkamide	Balanced	360 (CHO)	134	Internalization of	NQ	NA	[30]
	agonist			βarr2-GFP seen			
DAMGO	Balanced	40 (CHO)	100	Internalization of	NQ	5462471	[30]
	agonist			βarr2-GFP seen			
Herkinorin	Balanced	7.08 (Glosensor)	104	7.15 (BRET)	104	11431898	[24]
	agonist						
DAMGO	Balanced	8.58 (Glosensor)	100	8.3 (BRET)	100	5462471	[24]
	agonist						
Kurkinorin	G-protein	1.2 (cAMP)	100	140 (PathHunter)	96	132079904	[31]
DAMGO	Balanced	0.6 (cAMP)	100	42 (PathHunter)	100	5462471	[31]
	agonist						
1	G-protein	0.03 (cAMP)	100	14 (PathHunter)	81	NA	[32]
DAMGO	Balanced	0.6 (cAMP)	100	42 (PathHunter)	100	5462471	[32]
	agonist						
SR-11501	β-arrestin2	7.9(cAMP)	98	374 (PathHunter)	59	146025598	[33]
SR-17018	G-protein	76 (cAMP)	105	>10,000 (PathHunter)	10	130431397	[33]
DAMGO	Balanced	5.2 (cAMP)	100	229 (PathHunter)	100	5462471	[33]
	agonist						
SR-11501	β-arrestin2	133(GTPγS)	98	374 (PathHunter)	59	146025598	[33]
SR-17018	G-protein	193 (GTPγS)	72	>10,000 (PathHunter)	10	130431397	[33]
DAMGO	Balanced	34 (GTPγS)	100	229 (PathHunter)	100	5462471	[33]
	agonist						
SR-17018	G-protein	7.67 (cAMP)	62	6.48(BRET)	49	130431397	[26]
	partial agonist						
DAMGO	Balanced	8.48 (cAMP)	100	1200 (BRET)	100	5462471	[26]
	agonist						
2	G-protein	91(GTPγS)	74	>10,000 (PathHunter)	66	NA	[34]
3	G-protein	153 (GTPγS)	91	>10,000 (PathHunter)	12	NA	[34]
DAMGO	Balanced	34 (GTPγS)	100	229 (PathHunter)	100	5462471	[34]
	agonist						
DAMGO	Balanced	8.4 (cAMP)	100	6.7 (PathHunter)	100	5462471	[25]
	agonist						
MP102	G-protein	5.4 (cAMP)	88	5.2 (PathHunter)	16	NA	[25]
MP103	Balanced	6.5 (cAMP)	90	6.3 (PathHunter)	63	146025824	[25]
	agonist						
MP105	Balanced	6.7 (cAMP)	87	6.6 (PathHunter)	54	146025825	[25]
	agonist						

Assestment of G-protein and βarrestin-2 recruitment of ligands targeting MOR. G-protein biased ligands shown in bold along with control balanced agonist. G-protein biased ligands shown in bold along with control balanced agonist. NQ-not quantified; NA-not available.

**Table 2:** Ligands targeting the KOR.

Ligand	Functional	G-Protein	E_max_	β-Arrestin2	E_max_	PubChem	Ref
	Selectivity	EC_50_ (nM)		EC_50_ (nM)		ID	
RB64	G-protein	5.2 (cAMP)	99	1130 (Tango)	126	73347341	[59]
Salvinorin A	Balanced	4.73 (cAMP)	100	10.5 (Tango)	100	128563	[59]
	agonist						
Mesyl Salvinorin B	G-protein	0.12 (cAMP)	101	236 (PathHunter)	90	11271318	[60]
U50,488H	Balanced	0.23 (cAMP)	100	162 (PathHunter)	100	3036289	[60]
	agonist						
Triazole 1.1	G-protein	77 (GTPγS)	101	4995 (PathHunter)	98	46245518	[61]
U50,488H	Balanced	24 (GTPγS)	100	52.7 (PathHunter)	100	3036289	[61]
	agonist						
HS665	G-protein	4.98 (GTPγS)	88	463 (PathHunter)	55	71452041	[62]
HS666	G-protein	35.7 (GTPγS)	50	449 (PathHunter)	24	71452040	[62]
U69,693	Balanced	18.2 (GTPγS)	100	67.7 (PathHunter)	100	105104	[62]
	agonist						
6’GNTI	G-protein	1.6 (BRET)	64	Inactive (BRET)	NQ	146673012	[63]
U50,488H	Balanced	43 (BRET)	100	2000 (BRET)	100	3036289	[63]
	agonist						
6’GNTI	G-protein	2.1 (GTPγS)	37	5.9 (PathHunter)	12	146673012	[64]
U50,488H	Balanced	69 (GTPγS)	100	59 (PathHunter)	100	3036289	[64]
	agonist						
Isoquinolinone 2.1	G-protein	84.7 (GTPγS)	89	Inactive (PathHunter)	NQ	121231409	[65]
U69,693	Balanced	51 (GTPγS)	100	131 (PathHunter)	100	105104	[65]
	agonist						
Collybolide	G-protein	2 (GTPγS)	124 *	NA	NA	21669398	[66]
Salvinorin A	Balanced	0.2 (GTPγS)	136 *	NA	NA	128563	[66]
	agonist						

Assestment of G-protein and βarrestin-2 recruitment of ligands targeting KOR. G-protein biased ligands shown in bold along with control balanced agonist. * %Basal, NQ-notquantified, NA-not available.

**Table 3:** Ligands targeting the DOR.

Ligand	Functional	G-Protein	E_max_	β-Arrestin2	E_max_	PubChem	Ref
	Selectivity	EC_50_ (nM)		EC_50_ (nM)		ID	
PN6047	G-protein	8.9 (BRET)	128	145 (BRET)	115	121430051	[105]
DADLE	Balanced	2.5 (BRET)	100	69 (BRET)	100	6917707	[105]
	agonist						
2S-LP2	G-protein	32 (BRET)	93	1862 (BRET)	72	146025789	[106]
DADLE	Balanced	59 (BRET)	100	20 (BRET)	100	6917707	[106]
	agonist						
TAN-67	G-protein	2.5 (cAMP)	100	12.6 (PathHunter)	41	9950038	[107]
KNT-127	G-protein	2 (cAMP)	100	3.2 (PathHunter)	71	275705784	[107]
ARM390	G-protein	126 (cAMP)	100	316 (PathHunter)	103	9841259	[107]
DPDPE	Balanced	6.3 (cAMP)	100	25.1 (PathHunter)	100	104787	[107]
	agonist						
ARM390	G-protein	110 (BRET)	120	832 (BRET)	137	9841259	[105]
DADLE	Balanced	2.5 (BRET)	100	69 (BRET)	100	6917707	[105]
	agonist						
JNJ20788560	G-protein	5.6 (GTPγS)	92	NA	NA	46911863	[88]
SNC80	Balanced	5.4 (GTPγS)	100	NA	NA	123924	[88]

Assestment of G-protein and βarrestin-2 recruitment of ligands targeting DOR. G-protein biased ligands shown in bold along with control balanced agonist. NA-not available.

**Table 4:** Ligands targeting the NOP.

Ligand	Functional	G-Protein	E_max_	β-Arrestin2	E_max_	PubChem	Ref
	Selectivity	EC_50_ (nM)		EC_50_ (nM)		ID	
Ro 65-6570	G-protein	17 (BRET)	96	427 (BRET)	84	15512229	[130]
OFQ/N	Balanced	3.6 (BRET)	100	9.6 (BRET)	100	6324645	[130]
	agonist						
Ro 65-6570	G-protein	6.8 (BRET)	92	102 (BRET)	64	15512229	[133]
Cebranopadol	G-protein	3.2 (BRET)	86	Inactive (BRET)	NQ	11848225	[133]
OFQ/N	Balanced	6.9 (BRET)	100	6.6 (BRET)	100	6324645	[133]
	agonist						
MCOPPB	G-protein	0.025 (cAMP)	105	1585 (BRET)	99	24800108	[127]
SCH221,510	G-protein	4.3 (cAMP)	103	4266 (BRET)	87	9887077	[127]
NNC 63-0532	G-protein	26.3 (cAMP)	74	Inactive (BRET)	NQ	9803475	[127]
RTI-819	G-protein	72.4 (cAMP)	75	Inactive (BRET)	NQ	146034954	[127]
RTI-856	G-protein	7.24 (cAMP)	77	Inactive (BRET)	NQ	146034955	[127]
OFQ/N	Balanced	0.2 (cAMP)	100	204 (BRET)	100	6324645	[127]
	agonist						
BPR1M97	G-protein	1.8 (cAMP)	109	5100 (PathHunter)	14	137541784	[134]
OFQ/N	Balanced	0.4 (cAMP)	100	3 (PathHunter)	100	6324645	[134]
	agonist						

Assestment of G-protein and βarrestin-2 recruitment of ligands targeting NOP. G-protein biased ligands shown in bold along with control balanced agonist. NQ- not quantified.
